# Antioxidant Activity and Potential Cholesterol Modulating Effect of *Punica granatum* L. Peel Hydroethanolic Extract

**DOI:** 10.1002/cbdv.202501668

**Published:** 2025-11-08

**Authors:** Raja Chaaba, Amel Nakbi, Ilaria Rossi, Hanen Jrah, Zahra Amri, Nicola Ferri, Sonia Hammami, Stefano Dall'Acqua, Mohamed Hammami, Sounira Mehri

**Affiliations:** ^1^ LR12ES05, “Nutrition‐Functional Foods & Health” (NAFS), Faculty of Medicine University of Monastir Monastir Tunisia; ^2^ Higher School of Health Sciences and Technologies University of Sousse Sousse Tunisia; ^3^ Department of Medicine University of Padova Padova Italy; ^4^ Faculty of Pharmacy University of Monastir Monastir Tunisia; ^5^ Department of Pharmaceutical and Pharmacological Sciences University of Padova Padova Italy

**Keywords:** low‐density lipoprotein (LDL) receptor, oxidation, phenolic compounds, PCSK9, pomegranate peels

## Abstract

Lipoprotein metabolism is regulated by several key proteins, notably proprotein convertase subtilisin/kexin type 9 (PCSK9) and the low‐density lipoprotein receptor (LDLR). *Punica granatum* peel extract has been reported to exhibit lipid‐lowering properties; however, the underlying mechanisms of its action on lipid metabolism remain insufficiently characterized. In this study, the hydroethanolic extract of *P. granatum* peel (PPE) was found to be rich in phenolic compounds and exhibited strong antioxidant activity. Characterization by liquid chromatography coupled with diode array and multiple‐stage mass spectrometry (LC‐DAD‐MS*ⁿ*) identified punicalin, pedunculagin I, and galloyl‐HHDP‐hexose as the main constituents. Similar to statin treatment, PPE exposure significantly upregulated PCSK9 and LDLR protein expression in cultured cells compared to untreated controls, though the effect was less pronounced than that observed with statins. These findings suggest that phenolic‐rich PPE may serve as a promising natural source for the development of therapeutic agents aimed at preventing cardiovascular diseases.

## Graphical abstract

Punica granatum peel extract is rich in phenolic compounds, exhibits strong antioxidant activity, and modulates the expression of PCSK9 and LDL receptors. Collectively, these findings suggest a potential cardioprotective effect of the extract.

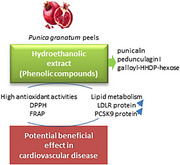

## 1 Introduction

*Punica granatum* L., commonly known as pomegranate and belonging to the Lythraceae family, is among the oldest fruit trees cultivated by humans. Pomegranate is a species native to Western Asia, but it has been cultivated and naturalized since ancient times in the regions of the Mediterranean basin. Pomegranate is an ancient and a popular fruit in Tunisia. The pomegranate tree is cultivated throughout the country, except in high‐altitude areas. Because of the excellent flavor, nutritional value, and medicinal properties of pomegranate, its cultivation has extended from a family character, with plantations in allotments, gardens, and/or scattered plantations, to public orchards, ensuring fruit distinction on a national scale. Pomegranate is a large round berry with a hard‐leathery bark, containing numerous arils in compartments delimited by thick partitions. Only the arils constitute the edible part of the pomegranate, representing approximately half of the fruit. The fruit is consumed fresh, and it is also used for the manufacture of juices, jellies, and jam. The by‐products, such as the fruit peels and seeds, are traditionally used in folk medicine and dyeing. They constitute a source of various bioactive molecules, such as phenolic compounds and flavonoids [1]. Indeed, the peels have potentially beneficial effects in diabetes, Alzheimer's disease, cancer, metabolic syndromes, and cardiovascular diseases (CVDs) [2, 3, 4, 5].

CVDs are the major cause of death in the world. They are the result of a disturbance in many mechanisms, such as inflammation, oxidation, lipid metabolism, hypertriglyceridemia, high levels of low‐density lipoprotein‐cholesterol (LDL‐C), and decreased levels of high‐density lipoprotein‐cholesterol (HDL‐C), that are usually associated with a high risk of CVDs. Currently, there are many therapeutic options for preventing CVDs, including the inhibitors of cholesterol synthesis (statins) or absorption (ezetimibe), the peroxisome proliferator activated receptors (PPAR)‐alpha receptor agonists (fibrates), and monoclonal antibodies against proprotein convertase subtilisin/kexin type 9 (PCSK9) [6].

Although these drugs offer an effective therapeutic option for controlling hypercholesterolemia, natural products, thanks to their extensive pharmacological and biological activities, which include lipid‐lowering, antioxidant, anti‐inflammatory, anti‐thrombotic, and immunomodulatory effects [7, 8], are considered valid alternatives for preventing CVDs, especially in primary prevention in patients at low risk. The phytoconstituents of natural products cover a diverse range of chemical entities, such as polyphenols, flavonoids, steroidal saponins, organosulfur compounds, vitamins, and polysaccharides [9, 10, 11]. Many studies were conducted, and they have reported that polyphenols are used to ameliorate dyslipidemia, especially to decrease LDL‐C level and oxidation [12]. Polyphenols act on cholesterol synthesis, metabolism, and uptake by inhibiting the hydroxy‐methyl glutaryl coenzyme A (HMG‐CoA) reductase, activating the cholesterol ester transfer protein (CETP), and upregulating low‐density lipoprotein receptor (LDLR) and downregulating PCSK9 expressions, respectively [12, 13].

PCSK9, discovered 20 years ago [14], is expressed particularly in the liver, intestinal epithelium, kidney, and the neurons. It plays a crucial role in LDL‐C assimilation by the liver. In the absence of PCSK9, circulating LDL particles are recognized by the LDLR, and the LDL–LDLR complex is then internalized by endocytosis. In the endosomes, the LDLs are dissociated from the receptors that will be recycled to the surface of the cells [15]. In the presence of PCSK9, recycling of LDLR is disrupted, leading to an increased LDL in the plasma. Other studies have demonstrated that PCSK9 can act on the hepatic production of triglycerides and on the metabolism of HDL [16, 17]. So, PCSK9 inhibitors are used to prevent or treat dyslipidemia. Many biotechnological strategies have been developed to reach this target [18]. Monoclonal antibodies and small interfering RNA (siRNA) are now available, and macrocyclic peptides against PCSK9 are under development [19]. However, many studies have focused on natural products [20, 21, 22, 23, 24] that can affect PCSK9 expression to identify new orally absorbed small molecules [13]. Polyphenols are among these natural products. It is for these reasons that foods rich in phenolic compounds, like *P. granatum* [1], could be of great benefit in preventing dyslipidemia and CVDs.

Many parts of pomegranate have been used, namely, the flowers, leaves, juice, and peels. Pomegranate peels have many beneficial health effects. Indeed, they are known for their anti‐inflammatory, antiapoptotic, and antioxidant effects [24]. These effects are the result of the pomegranate's richness in phenolic compounds, mainly ellagitannins [25, 26]. Peel extracts are known also to have lipid‐lowering effects by decreasing total lipids, total cholesterol, and LDL‐C and increasing HDL‐C [27]. It is for this reason that Sadeghipour et al. suggested that further studies are needed to explore the effect of pomegranate peels in dyslipidemia [28]. According to our knowledge, the mechanisms by which pomegranate peels intervene in lipid metabolism are not yet well described.

The objective of the present study was to elucidate the effect of pomegranate peel extract (PPE) on the expression of PCSK9 and LDLR in hepatic carcinoma cells (Huh7) to better understand its influence on lipid metabolism. Additionally, the chemical composition (using spectrophotometric methods and LC‐DAD‐MS*^n^*) and antioxidant activities (radical scavenging and reducing power) of the extract were characterized to support its potential valorization.

## 2 Results and Discussion

### 2.1 Extract Characterization

Total phenolic compound and flavonoid concentrations in PPE are summarized in Table 1. The extract was found to be very rich in phenolic compounds (202.62 ± 4.75 mg gallic acid equivalent/g extract) and in flavonoids (79.52 ± 0.90). It also had a powerful antioxidant activity (Table 1). The antioxidant activity was evaluated by free radical scavenging assay and reducing power. The concentrations providing 50% of radical scavenging activity and reducing power inhibition were very low, demonstrating a high antioxidant activity for PPE. With regard to 2,2‐diphenyl‐1‐picrylhydrazyl (DPPH) essay, EC_50_ of PPE was only 0.304 mg/mL. EC_50_ obtained from reducing power essay was 0.385 mg/mL.

**TABLE 1:** Total phenolic compounds, total flavonoids, and EC_50_ (mg/mL) values obtained from 2,2‐diphenyl‐1‐picrylhydrazyl (DPPH) antioxidant assay and reducing power of *Punica granatum* peel extract.

	Pomegranate peel extract
Total phenolic compounds (mg gallic acid equivalent/g extract)	202.62 ± 4.75
Total flavonoids (mg catechin equivalent/g extract)	79.52 ± 0.90
EC_50_ (mg/mL) DPPH	0.304 ± 0.008
EC_50_ (mg/mL) reducing power	0.385 ± 0.039

Phenolic compounds have been largely employed to enhance the treatment of CVDs [29]. Peel extracts derived from pomegranate fruits are notably abundant in both total phenolic compounds and flavonoids [1]. However, compared with many studies, our extract exhibited a higher proportion of flavonoids, which may be attributed to the extraction solvent employed. Methanol, aqueous [26], and hydro‐methanol [30] peel extract showed less flavonoid proportion than hydro‐ethanolic extract [31]. In the present study, ethanol/water extraction was used due to the higher concentration of these compounds in this phase. Javani‐Seraji et al. showed that the ethanolic extract (50%) exhibits the greatest total phenolic content compared to methanol, acetone, and water extracts [32].

In the present study, PPE had high phenolic compounds and flavonoid concentrations. It also had a high antioxidant activity (measured by DPPH free radical scavenging activity and reducing power essay methods). Indeed, the antioxidant activity and the phenolic content are usually strongly correlated [33]. DPPH free radical scavenging activity informs us about the presence of molecules that neutralize free radicals (DPPH). Reducing power essay gives us an idea about the presence of reductones that break the free radical chain or the peroxide formation [34].

#### 2.1.1 LC‐DAD‐MS*^n^*

The analysis revealed that the hydroethanolic extract presented several constituents that could be ascribed to pomegranate phytochemicals. For instance, the chromatogram recorded with diode array detector (DAD) at 254 nm presented several peaks with UV spectra resuming tannins correlated to punicalagin and ellagic acid derivatives and the presence of flavonoid. The MS fragmentation obtained in negative ion mode and the comparison with reference compounds allowed the identification of punicalagin, rutin, and ellagic acid that were also confirmed by standard injection. Furthermore, other constituents were tentatively identified through MS data. The LC‐DAD‐MS*^n^* analysis revealed a complex profile with a predominance of hydrolyzable tannins, including punicalin, punicalagin α and β, pedunculagin I, punigluconin, galloyl‐HHDP‐hexose, and granatin B, along with the flavonoid rutin and phenolic derivatives such as ellagic acid and its glycosylated forms (hexoside and deoxyhexoside) (Table 2). Punicalin (10.74 ± 0.91 mg/100 mg) was the most abundant constituent, followed by pedunculagin I (5.26 ± 0.23 mg/100 mg) and galloyl‐HHDP‐hexose (4.13 ± 0.43 mg/100 mg).

**TABLE 2:** HPLC‐DAD‐MS*^n^* identification and quantification in mg/100 mg of *Punica granatum* peel extract.

RT (min)	Compound	M − H	Fragments	mg/100 mg
1.4	Punicalin	781	601 721 299 271	10.74 ± 0.91
3.29	Punicalagin α^a^	1083	601 781 299 271	0.02 ± 0.01
4.1	Punicalagin β^a^	1083	601 781 299 271	2.25 ± 0.10
4.5	Pedunculagin I	783	481 301	5.26 ± 0.23
4.5	Punigluconin	799	479 273 313 417	1.33 ± 0.14
5.2	Galloyl‐HHDP‐hexose	633	301 461 229 257 185	4.13 ± 0.43
5.5	Ellagic acid hexoside	463	301 229 257 185 201	3.12 ± 0.23
6	Granatin B	951	897 445 613 729 401 229	1.17 ± 0.11
6.7	Rutin^a^	609	301 271	0.30 ± 0.05
6.9	Ellagic acid^a^	301	229 257 185 201	0.09 ± 0.02
6.9	Ellagic acid hexoside	463	301 229 257 185 201	0.05 ± 0.01
7.6	Ellagic acid deoxyhexoside	447	301 229 257 185 201	2.38 ± 0.12

^a^Compared with reference standard.

Hydrolyzable tannins are polyphenolic substances derived from gallic acid (3,4,5‐trihydroxybenzoic acid), including gallotannins and ellagitannins. Similarly, other studies have shown that ellagitannin is dominant in pomegranate peels [25, 35]. The most abundant type of ellagitannin is punicalagin [36]. However, our results showed that punicalin was the most abundant one. Punicalagin is the most powerful antioxidant compared to other phenols [37]. The extract contains another hydrolysable tannin. It is galloyl‐HHDP‐hexose, which belongs to the gallotannin group. Both ellagitannin and gallotannin have gallic acid as molecule precursor. Collectively, the observed quantitative pattern indicates a distinctive phenolic fingerprint, characterized by high punicalin content, moderate punicalagin levels, and substantial diversity in hydrolyzable tannins, potentially contributing to the extract's strong antioxidant and bioactive potential.

#### 2.1.2 Cell Viability

**FIGURE 1:**
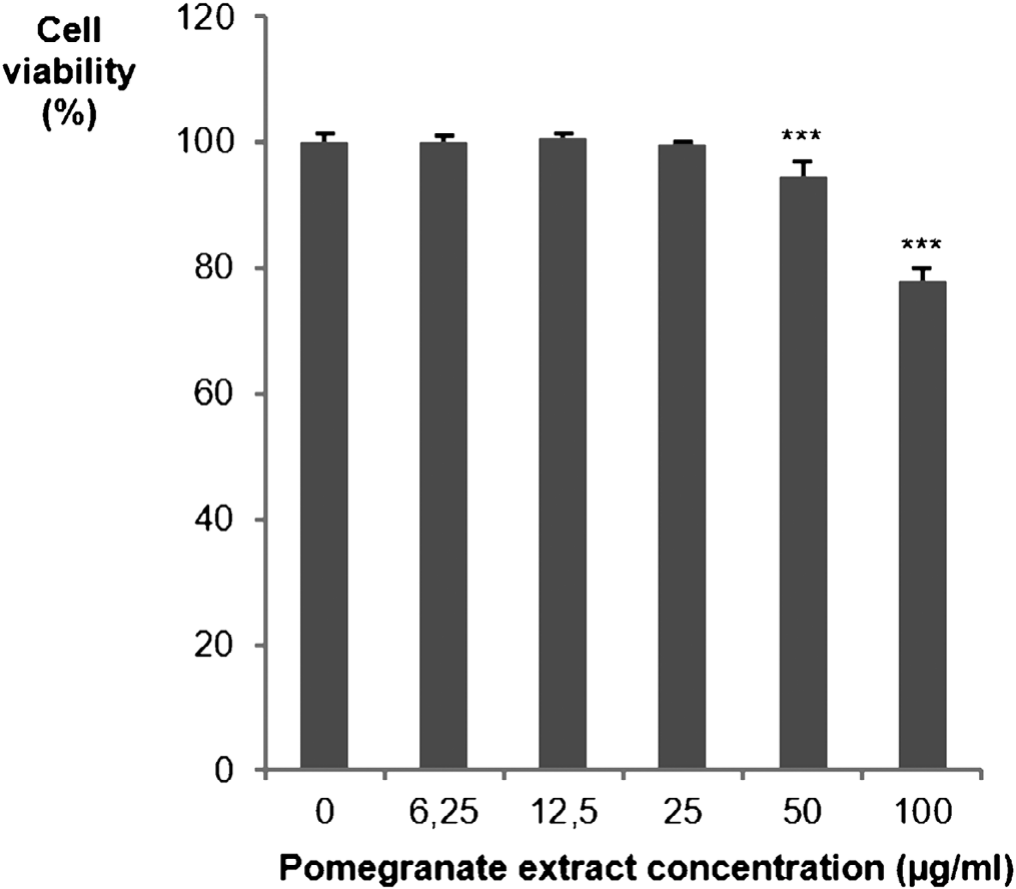
Effect of PPE on hepatoma cell line Huh7 viability. The cytotoxic effect was determined by SRB assay after a 72‐h incubation of Huh7 with indicated concentrations of the extract. Data are expressed as mean ± SD of three independent experiments. *p < 0.05; **p < 0.01; ***p < 0.001 versus untreated controls by Student's *t*‐test.

Before examining the effect of PPE on *LDLR* and *PCSK9* gene expression, the maximum nontoxic concentration in Huh7 cells was determined. As shown in Figure 1, PPE begins to significantly reduce cell viability at 50 µg/mL, indicating a cytotoxic effect at higher doses. Therefore, subsequent experiments were performed at concentrations below this threshold (25 µg/mL) to avoid interference from cell death. PPE has demonstrated cytotoxic effects on hepatic carcinoma cells, notably the HepG2 cell line. The cytotoxicity is dose‐dependent. PPE has been shown to reduce cell viability, induce apoptosis, and cause cell cycle arrest in the G0/G1 and S phases [38].

### 2.2 Effect of PPE on *PCSK9* and *LDLR* Expressions

**FIGURE 2:**
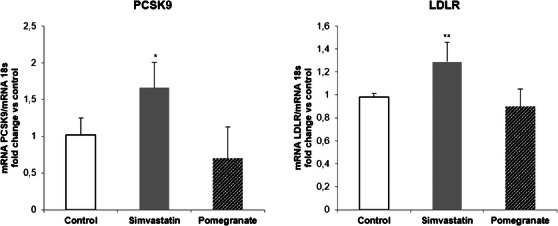
Effect of PPE on LDLR and PCSK9 mRNA expressions in Huh7 cell line. Cells were incubated with simvastatin (20 µM) and PPE (25 µg/mL) for 24 h. At the end of the incubation, total RNA were extracted and LDLR and PCSK9 mRNA levels were determined by quantitative real‐time PCR. *p < 0.05; **p < 0.01 versus controls by Student's *t*‐test. LDLR, low‐density lipoprotein receptor; PCSK9, proprotein convertase subtilisin/kexin type 9.

Huh7 cells were incubated with noncytotoxic concentrations of PPE (25 µg/mL) for 24 h. As positive control, simvastatin 20 µM (final concentration) was used. The results were compared to the untreated cells (control). The quantification of mRNA levels showed that both *PCSK9* and *LDLR* expressions were induced by simvastatin, whereas PPE showed no effect (Figure 2).

**FIGURE 3:**
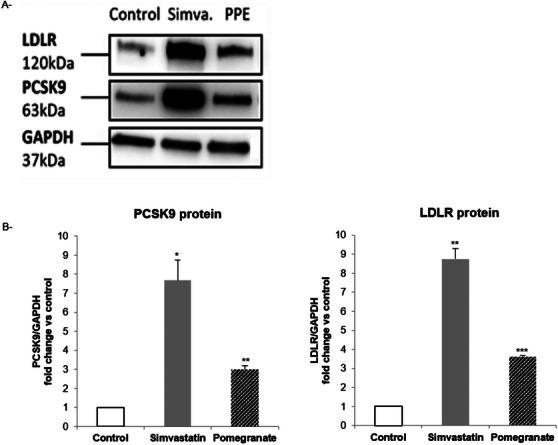
Effect of PPE on LDLR and PCSK9 protein expressions in Huh7 cell line. Cells were incubated with simvastatin (20 µM) and PPE (25 µg/mL) for 72 h. At the end of the incubation, total proteins were extracted and LDLR and PCSK9 levels were determined by western blot analysis. GAPDH was used as loading control (A). Densitometric readings were evaluated using the Image Lab software and relative intensities were shown in the histograms (B). *p < 0.05; **p < 0.01; ***p < 0.001 versus controls by Student's *t*‐test. LDLR, low‐density lipoprotein receptor; PCSK9, proprotein convertase subtilisin/kexin type 9; Simva, simvastatin.

Western blot analysis was used to assess the effect of simvastatin and PPE on protein expressions of LDLR and PCSK9. As expected, simvastatin significantly increased both PCSK9 and LDLR protein levels (Figure 3). A positive induction of both proteins was also observed after incubation with PPE.

Phenolic compounds are known for their benefits in CVDs given their effects on several mechanisms, such as oxidation, signaling cascade, and endothelium dysfunction [39]. Moreover, polyphenols intervene by acting on lipid metabolism [40]. Many in vivo and in vitro investigations elucidated the pomegranate phenolic compounds effect on improving lipid profile [28, 41]. In Huh7 cell, Khateeb et al. showed that pomegranate polyphenols upregulate the expression and activity of paraxonase1 and paraxonase2 that protect lipoproteins from oxidation [42]. This effect could also be due to an upregulation of ATP binding cassette subfamily A1 and liver X receptor α, which enhances cholesterol efflux in macrophages [43] or the activation of the PPARγ‐ABCA1/CYP7A1 cell signaling pathway in human hepatocyte cell line L‐02 [44]. In hamsters, ellagic acid promotes cholesterol removal by increasing fecal bile acid and upregulating the mRNA levels of the two pathways, LXR (/PPAR‐ABCA1) [45]. In western diet‐fed male mice, punicalagin modulates lipid intake and clearance in the liver [46]. However, few studies have explored the effect of pomegranate on PCSK9 and LDL receptors. Indeed, as PCSK9 and LDLR play a main role in cholesterol clearance, many studies have been conducted to explore the effect of different plant extracts on this pathway [22, 23, 47]. Dehghani et al. showed that PPE and punicalagin significantly increased LDLR protein levels and LDL cholesterol uptake and exhibited a dose‐dependent reduction in the expression of PCSK9 in HepG2 cells [48]. In our study, the incubation of Huh7 cells with statins was associated with increased concentrations of LDLR and PCSK9 mRNA. However, PPE has no significant effects on mRNA levels of these targets. In contrast, statins and PPE are associated with increased PCSK9 and LDLR protein levels. PCSK9 and LDLR have the same SRE (sterol regulatory element) motif that acts as promoter. Statins exert an upregulatory influence on both PCSK9 and LDLR expressions via sterol regulatory element binding proteins (SREBP). This effect is mediated by the ability of statins to enhance the binding of SREBP2 to SRE‐1, thus reducing plasma cholesterol levels [49]. Our results did not show any significant effect of PPE on mRNA of *PCSK9* and *LDLR* genes. However, an important increase was demonstrated with regard to the protein level. The potential mechanism of action may involve either the activation of translation or the intracellular accumulation of both proteins (PCSK9 and LDLR). This can be explained by the fact that certain compounds in the extracts (flavonoids or polyphenols) may inhibit the proteasomal or lysosomal degradation of PCSK9 and LDLR by modulating the activity of proteolytic enzymes, thereby increasing their half‐life and accumulation. Additionally, some plant metabolites may directly bind to PCSK9 or LDLR, altering their conformation and protecting them from degradation. This can also affect their detection during analyses (e.g., improved antibody recognition in western blot). Furthermore, the extract may act on proteins that regulate the stability of PCSK9 or LDLR, such as IDOL (inducible degrader of the LDLR) or ubiquitin ligases, without altering the transcription of the *PCSK9* and *LDLR* genes. Further studies are needed to better understand the mode of action of PPE. The upregulation of LDLR correlates with enhanced clearance of circulating LDL cholesterol, resulting in decreased plasma cholesterol levels and offering protection against CVDs. In contrast, increased PCSK9 expression promotes LDLR degradation and reduces receptor recycling, which elevates circulating LDL cholesterol levels. The potential protective role of elevated PCSK9 in CVD remains controversial. In the present study, the modest increase in PCSK9 suggests a homeostatic mechanism to prevent excessive LDL‐C clearance that could disrupt essential cellular functions. Moreover, emerging evidence indicates that PCSK9 possesses pleiotropic effects beyond LDLR regulation, including modulation of inflammatory pathways, vascular homeostasis, and immune responses [50]. Therefore, a moderate elevation of PCSK9 may contribute to cardiovascular protection by balancing these complex biological processes.

## 3 Conclusions

Due to their antioxidant properties and their effects on PCSK9 and LDLR, the phenolic‐rich extract of pomegranate (PPE) appears to represent valuable resources for the development of molecules to prevent CVDs. However, it is imperative that a comprehensive series of additional studies be conducted not only to identify and isolate the specific bioactive compounds within these extracts but also to gain a deeper understanding of the intricate mechanisms underlying their mode of action. Such investigations will contribute significantly to the advancement of knowledge regarding the potential therapeutic applications of PPE in the context of cardiovascular health.

## 4 Experimental Section

### 4.1 Extract Preparation and Characterization

#### 4.1.1 Extract Preparation

Pomegranate fruits were harvested in December 2023 from the Sousse region (35°49′32″ N, 10°38′28″ E) in central Tunisia. They were identified as *P. granatum* L. by Professor Mohamed Chaib from the Faculty of science, Sfax University, Tunisia, and a voucher specimen (Voucher No. L02‐2025) was deposited in the herbarium at the Faculty of science, Sfax University, The fruits were peeled off by hand and the peels were freeze dried (lyophilized) and blended. The powdered peels were extracted by hydroethanolic solutions [ethanol/water (40/60, v/v)] at a solvent‐to‐peel powder ratio of 10:1 (w/v) during one night with stirring. Then, the mixture was filtered under vacuum and the filtrate was subjected to rotary evaporation at 40°C.

#### 4.1.2 Total Phenolic Content

The dosage of total phenolic compounds was carried out using the colorimetric Folin–Ciocâlteu method according to Montedoro et al. [51], with slight modifications [1]. Gallic acid was used to prepare standard range and to determine the calibration curve equation. The results were expressed as mg gallic acid equivalents per g of extract.

#### 4.1.3 Total Flavonoid Content

The dosage of flavonoids was estimated by the colorimetric method of aluminum trichloride (AlCl_3_) at 510 nm [52]. The results were expressed as mg catechin equivalent per g of extract by referring to a calibration curve produced with catechin.

#### 4.1.4 In Vitro Antioxidant Activity

The antioxidant activity of the two extracts was evaluated using two colorimetric methods: DPPH free radical scavenging activity and the reduction of ferric iron by the FRAP method (ferric reducing antioxidant power).

##### 4.1.4.1 DPPH‐Free Radical Scavenging Activity

DPPH, a free radical of violet color in solution, was reduced to 2,2 diphenyl 1 picryl hydrazine of yellow color by a free radical sensor, and it showed a characteristic absorbance at a wavelength of 517 nm. DPPH free radical scavenging activity was calculated by following DPPH absorbance in the absence and in the presence of the extract. A volume of 1 mL of the DPPH solution was added to different quantities of extract. The mixtures were shaken and kept in the dark for 30 min. Finally, the optical density was measured at a wavelength of 517 nm using a spectrophotometer, with reference to a control sample without extract. The measured absorbance was then converted into percentage inhibition relative to the absorbance of the control solution. The relationship between percentage inhibition and total phenolic content was not linear but logarithmic. The kinetic analysis of this activity allowed the determination of concentrations corresponding to 50% inhibition (IC_50_). The result was expressed as the concentration of the extract responsible for 50% of the radical scavenging activity (EC_50_) [53].

##### 4.1.4.2 Reducing Power

Measurement of reducing power by iron reduction was based on the increase in optical absorbance at 700 nm. To 250 µL of the extract solution, 625 µL of phosphate buffer solution (200 mM; pH 6.6) and 625 µL of 1% potassium ferricyanide [K_3_Fe(CN)_6_] were added. After incubation for 20 min at 50°C, 625 µL of 10% trichloroacetic acid (TCA) was added to the mixture, followed by centrifugation at 3000 rpm for 10 min. To 625 µL of the supernatant, 625 µL of distilled water and 125 µL of 0.1% ferric chloride (FeCl_3_) were added. The absorbance was measured at 700 nm, and the results were expressed based on the increase in optical density, which corresponds to an increase in the reducing power of the extracts.

The reducing power was calculated as a percentage of reduction. Then, the graph of reducing power percentage according the extract concentration was drawn. The result was expressed as the extract concentration responsible of 50% of reducing power (EC_50_) [54].

#### 4.1.5 Liquid Chromatography Diode Array Multiple Stage Mass Spectrometry, LC‐DAD‐MS*^n^*

A 20 mg of the extract was carefully weighed and dissolved in 10 mL of methanol. The solution was sonicated for 15 min, followed by centrifugation for 10 min, and the supernatant was collected for analysis.

The chemical characterization of the extracts was performed using an Agilent 1260 system, coupled with a 1260 DAD and a Varian MS 500 ion trap mass spectrometer. An SBC18 column (4.6 × 50 mm, 1.8 µm) was utilized for separation, with the mobile phases consisting of water (1% formic acid) (A), acetonitrile (B), and methanol (C). The elution gradient followed these conditions: 95:5:0% (A/B/C) at 0 min; 50:35:15% (A/B/C) at 10 min; 5:85:10% (A/B/C) at 16 min; 0:85:15% (A/B/C) from 17.0 to 17.5 min, followed by a 5‐min re‐equilibration phase. The flow rate was set to 0.7 mL/min, with an injection volume of 10 µL. Chromatograms were obtained using the DAD within the 200–600 nm range, with traces recorded at 254, 280, 330, and 590 nm. UV spectra were acquired to identify each peak. Mass spectra were acquired using a mass spectrometer with an electrospray ionization source (EIS) in negative ion mode, covering a mass range of 105–2000 *m*/*z*. The ion trap acquired data in TDDS mode, enabling multiple reactions monitoring with multistage fragmentation. This approach allowed for the identification of secondary metabolites through comparison with reference standards and published literature. The mass spectrometer parameters were as follows: needle voltage set to 4100 V, nebulizer gas pressure at 30 psi, drying gas temperature at 270°C, drying gas pressure at 25 psi, spray chamber temperature at 50°C, capillary voltage at 75 V, and RF loading at 70%.

Chlorogenic acid, rutin, ellagic acid, and mulberroside A were used for quantification. Calibration curves were generated using standard solutions within the range of 1–50 µg/mL.

### 4.2 In Vitro Experiment

#### 4.2.1 Cell Culture and Treatment

Huh7 cell lines were purchased from Tebubio SRL, Milan, Italy (code product 300156). All cell culture maintaining reagents and all the plastic supplies were obtained from EuroClone (Milan, Italy). The extracts were diluted in dimethyl sulfoxide (DMSO, Sigma‐Aldrich). A stock solution (50 mM, pH 7.2) of simvastatin (Merck, Sharp, and Dohme Research Laboratories, Kenilworth, NJ, USA) was prepared. Culture medium was MEM supplemented with 10% fetal bovine serum (FBS), 1% sodium pyruvate 100×, 1% l‐glutamine 200 mM, 1% penicillin/streptomycin solution, and 1% nonessential amino acids 100×. Culture conditions were 37°C, 5% CO_2_, and 95% air.

For the experiments, human hepatic cancer cells (Huh7) were incubated in MEM/10% FBS with the final concentration of DMSO did not exceed 0.5% v/v.

#### 4.2.2 *PCSK9* and *LDLR* Expression

Cells were seeded at a density of 80 000 cells, using MEM supplemented with 10% FBS. After 24 h, treatments were added. After 24 h, the cells were rinsed twice with PBS. Then, the iScript RT‐qPCR sample prep reagent (Bio‐Rad, Milan, Italy) was used to extract total RNA. Specific primers for *18S* (FWD 5′‐CGGCTACCACATCCACGGAA‐3′, REV 5′‐CCTGAATTGTTATTTTTCGTCACTACC‐3′), *PCSK9* (FWD 5′‐CCTGCGCGTGCTCAACT‐3′, REV 5′‐GCTGGCTTTTCCGAATAAACTC‐3′), and *LDLR* (FWD 5′‐TCTATGGAAGAACTGGCGGC‐3′ REV 5′‐ACCATCTGTC TCGAGGGGTA‐3′) were used for qPCR. The analyses were carried out using the following program: 10 min at 45°C, 5 min at 95°C, and 40 cycles of 5 s at 95°C, followed by 30 s at 60°C. The data were presented as *Ct* values and utilized for relative quantification of targets using ΔΔ*Ct* calculations. The ΔΔ*Ct* values were calculated by multiplying the ratio of the efficiency of specific primers to that of the housekeeping gene 18S. The efficiency was determined using the formula ((10^(−1/slope)^) − 1) × 100 [55].

#### 4.2.3 Western Blot Analysis

Anti‐PCSK9, anti‐LDLR, and anti‐GAPDH primary antibodies (rabbit polyclonal antibody, GeneTex) were used. Anti‐rabbit secondary antibody was obtained from Jackson Immuno Research.

Cells were plated at a density of 300 000 cells per well in MEM/10% FBS. After 24 h, treatments were added. Seventy‐two hours later, cells were subjected to two washes with PBS (Sigma‐Aldrich) and were homogenized in lysis buffer (1% NP‐40, 50 mM Tris–HCl and 150 mM NaCl, pH 7.5). BCA assays (SERVA) were used to assess protein concentration. Then, 25 µg of total protein extract per sample were loaded onto a 4%–20% SDS–PAGE gel (Bio‐Rad) and separated under denaturing and reducing conditions. Following electrophoresis, the proteins were transferred to a nitrocellulose membrane. Blocking was carried out with 5% nonfat dried milk in tris‐buffered saline containing 0.2% Tween 20 (TBST20). Nitrocellulose membrane with the primary antibodies was incubated overnight at 4°C in agitation. Membrane and horseradish peroxidase conjugated secondary antibodies were incubated at room temperature for 1.5 h under agitation. UVITEC Alliance Q9 Advanced—manual system was used to measure luminescence signals and ImageJ software was used for quantitative densitometric analysis.

### 4.3 Statistical Analysis

It was conducted with SPSS 21.0 for Windows (SPSS, Chicago, IL, USA). Data are expressed as mean ± standard deviation. Comparison between means was performed using Student's *t*‐test. A *p* value <0.05 was considered statistically significant.

## Author Contributions

**Raja Chaaba**: conceptualization, laboratory analysis, writing original draft, review and editing of the final manuscript. **Amel Nakbi**: conceptualization. **Ilaria Rossi**: laboratory analysis, review and editing of the final manuscript. **Hanen Jrah**: writing original draft. **Zahra Amri**: laboratory analysis. **Nicola Ferri**: supervision, review and editing of the final manuscript. **Sonia Hammami**: project administration. **Stefano Dall'Acqua**: supervision, review and editing of the final manuscript. **Mohamed Hammami**: supervision. **Sounira Mehri**: data analysis.

## Conflicts of Interest

The authors declare no conflicts of interest.

## Funding

The authors received no specific funding for this work.

## Data Availability

Data are available on request from the authors.
